# Prevalence of Vitamin D Deficiency in Children with Type 1 Diabetes Mellitus

**DOI:** 10.7759/cureus.7836

**Published:** 2020-04-26

**Authors:** Mauri Carakushansky, Priya Patel, Bertha A Ben Khallouq, Shilpa Gurnurkar

**Affiliations:** 1 Pediatric Endocrinology, Nemours Children's Hospital, Orlando, USA; 2 Internal Medicine, University of Mississippi Medical Center, Jackson, USA; 3 Medicine, University of Central Florida, Orlando, USA

**Keywords:** vitamin d deficiency, vitamin d insufficiency, sun rich environment, type 1 diabetes mellitus

## Abstract

Background

In the recent years, controversy has emerged regarding the relationship between vitamin D deficiency and the potential effects it could have on glycemic control in patients with type 1 diabetes mellitus (T1D). This study investigates the prevalence of vitamin D insufficiency/deficiency in pediatric patients with T1D from a single, large volume practice.

Methods

This was a retrospective chart review that collected clinical/demographic data as well as serum 25(OH) D levels from medical records of 395 children between the ages of 3 and 18 years with T1D followed at Nemours Children’s Hospital. This data was compared to the National Health and Nutrition Examination Survey (NHANES) database. A Pearson’s Chi-square test was used between group associations. All statistical tests were two-sided and p < 0.05 was used for statistical significance.

Results

Of the 395 children included in these analyses, 4% were vitamin D deficient and 60% were vitamin D insufficient. There were no significant associations of vitamin D deficiency based on sex and age. Vitamin D deficiency was more common among White children when compared to Hispanic children and African American children (42% vs 29%; p < 0.001). Of those that were vitamin D insufficient (n = 235), most were Hispanic (51%), 36% White and 13% African American. There was a significant association between vitamin D deficiency and body mass index (BMI) (p = 0.035). In the summer, children were less likely to be vitamin D deficient (3% vs 6% in winter) and less likely to be vitamin D insufficient (55% vs 71% in winter) (p = 0.007).

Conclusions

Vitamin D insufficiency is highly prevalent among pediatric type 1 diabetics of Central Florida and statistically significant correlation was found between vitamin D status and ethnicity, BMI as well as seasonal variation.

## Introduction

Vitamin D deficiency is prevalent in both developed and developing countries. The presence of high affinity vitamin D receptors (VDR) in a variety of cell types has led researchers to believe that vitamin D deficiency could contribute to environmentally driven pathogenesis of many diseases, including type 1 diabetes mellitus (T1D) [1].

A link between autoimmune conditions and vitamin D deficiency/insufficiency has been implicated. A longitudinal study in Western Australia found both geographical and seasonal variations in incidence of T1D in children [2]. A recent cross-sectional study of Israeli type 1 diabetic youth revealed high prevalence of vitamin D insufficiency and an association between vitamin D levels, seasonality and BMI [3]. A similar study conducted in the United States also found a high prevalence of vitamin D inadequacy in youth with T1D [4]. Not surprisingly, according to a recent study by Lipman et al., both the incidence of T1D and vitamin D deficiency are on the rise [5].

A prospective study in Finland followed over 10,000 children for up to 31 years and concluded that those who received a daily dose of 2000 IU of vitamin D during infancy had a 78% risk reduction in developing T1D [6]. Vitamin D deficiency is associated with increased inflammatory markers in diabetics including C-reactive protein (CRP), monocyte toll-like receptor 2 (TLR-2), TLR-4, and nuclear factor-κB (NFκB) expression which might predict increased risk of microvascular complications. Some studies have looked at possible links between vitamin D status and diabetes complications such as nephropathy and retinopathy. In people without diabetes, some studies have found a link between low vitamin D levels and increased levels of proteinuria, a sign of nephropathy. Others have suggested that drugs which improve the body’s ability to utilize vitamin D may be helpful in the treatment of chronic kidney disease [7,8]. Another study has even suggested increased risk of death in patients with type 1 diabetes and untreated vitamin D deficiency [9]. Recently, a study revealed that type 1 diabetes is associated with an increased risk of fractures beginning in childhood [10]. Therefore, it may be possible that the significant prevalence of vitamin D insufficiency in type 1 diabetics might be a contributing factor in the development of decreased bone mineral density.

Diabetes is one of the fastest-growing chronic diseases worldwide. The number of U.S. children living with type 1 diabetes has increased by almost 60 percent since 2002, and experts are unsure of the cause. Using a national database, researchers found that the prevalence of type 1 diabetes stood at just under 1.5 cases per 1,000 children and teenagers in 2002. By 2013, that figure had risen to 2.3 per 1,000. This rise has also been accompanied by an increase in diabetes comorbidities [11]. As vitamin D deficiency also has become significantly more common, there may be an association between unrecognized vitamin D deficiency and the puzzling escalating incidence of type 1 diabetes and its comorbidities. There is also some epidemiologic evidence that decreased vitamin D level in pregnancy or early childhood may be associated with diabetes risk, but the evidence is not yet conclusive and additional studies are needed [12].

Our study estimates the prevalence of vitamin D deficiency/insufficiency in pediatric patients followed in our practice with type 1 diabetes mellitus and its association, if any with sociodemographic factors, BMI and diabetes control.

## Materials and methods

A retrospective chart review of children with T1D treated at the Nemours Children’s Hospital outpatient endocrine clinic between January 2016 and December 2018 (N = 395) was conducted utilizing electronic medical records. Sample was identified via ICD-10 codes (E10.9 and E10.65) and lab orders for serum 25 (OH) vitamin D. Children between the ages of 3 and 18 were included. Children with severe pathological risk factors associated with vitamin D deficiency, such as osteogenesis imperfecta, kidney disease or cystic fibrosis were excluded. Only those not already on vitamin D supplementation were included. Children younger than 3 were excluded to minimize the influence of extended breastfeeding on their initial vitamin D status. Additionally, there are very few children under the age of 3 with T1D in our practice. Including ages 3-18 enhanced the data homogeneity of our study population. This study was reviewed and approved by the Institutional Review Board at Nemours Children’s Hospital (IRB# 762598).

Serum 25 (OH) D and HbA1c

Serum 25 (OH) D assays were processed by a local laboratory utilizing liquid chromatography tandem mass spectrometry assay (LC-MS/MS). In order to control for effects of vitamin D supplementation, the 25 (OH) D levels before supplementation were used. A point of care (POC) hemoglobin A1C (HbA1C) level closest in time to the 25 (OH) D test was used. The POC HbA1C system, while providing immediate test results and quick therapy judgments, is subject to the same competency requirements as laboratory HbA1c methods and therefore provides comparable analytical presentation of bias, reliability, and error.

Statistical analysis and data management

In accordance with the national population represented in the NHANES 2001-2004 study (National Health and Nutrition Examination Survey), vitamin D levels were stratified into three groups: vitamin D deficiency (<15 ng/mL), vitamin D insufficiency (15-29 ng/mL), and vitamin D sufficiency (≥30 ng/mL) [13].

Gender, race/ethnicity, HbA1c status and BMI are reported as frequency (n) and percentage (%). In this study, race/ethnicity was defined as follows: Hispanics (Hispanic/Latin American descent), African Americans (non-Hispanic Blacks or African-Americans) and Caucasian (non-Hispanic Whites).

HbA1c and BMI were dichotomized as follows: HbA1c (adequate and suboptimal), where <9% is adequate and ≥9% suboptimal. BMI (obese and not obese) where obese ≥95th percentile and not obese <95th percentile. Age at which serum 25 (OH) D was obtained is reported as mean (SD) and median (min-max); to test for mean age differences a one-way ANOVA was used.

A Pearson’s Chi-square was used between group associations: First, associations of vitamin D levels (deficient, insufficient and sufficient) and sociodemographic factors (gender, race/ethnicity and age) were conducted. As a second step, associations of vitamin D levels and HbA1c and BMI were conducted. As a final step, the same associations, separately for children that had serum drawn in the summer months (March-October) and the winter months (November-February) were conducted. All statistical tests were two-sided and p < 0.05 was used for statistical significance.

## Results

Association of vitamin D deficiency and sociodemographic factors

There were 395 children included in these analyses: Four percent of children were vitamin D deficient, 60% of children were insufficient, and 36% were sufficient (Table 1).

**Table 1:** Participant Characteristics by Vitamin D Levels When percentages do not add to 100%, it is due to rounding. HbA1c: Hemoglobin A1c; BMI: Body mass index.

	Vitamin D deficient <15 ng/mL (n = 17)	Vitamin D insufficient 15-29 ng/mL (n = 235)	Vitamin D sufficient ≥30 ng/mL (n = 143)	Total	P value
Sex					
Female	8 (47)	117 (50)	68 (48)	193 (49)	0.904
Male	9 (53)	118 (50)	75 (52)	202 (202)	
Race/Ethnicity					
African American	5 (29)	30 (13)	10 (7)	45	<0.001
Hispanic	5 (29)	120 (51)	46 (32)	171	
White	7 (42)	85 (36)	87 (61)	179	
Age					
Mean (SD)	14.60 (2.42)	12.96 (3.1)	12.64 (3.42)	12.91 (3.23)	0.057
Median (Min-Max)	14.72 (8-18)	13.06 (3-18)	13.04 (3-18)	13.17 (3-18)	
HbA1C Status					
Adequate	6 (35)	117 (50)	83 (58)	206 (52)	0.108
Suboptimal	11 (65)	118 (50)	42 (143)	189 (48)	
BMI Status					
Obese	5 (29)	24 (10)	13 (9)	42 (11)	0.035
Non-Obese	12 (71)	211 (90)	130 (91)	353 (90)	

There were no significant associations of vitamin D deficiency based on sex and age. There was a significant association between race/ethnicity and vitamin D levels. Specifically, vitamin D deficiency was more common among White children as compared to Hispanic children (42% vs 29%, respectively) and African American children (42% vs 29%). African American and Hispanic children were vitamin D deficient at the same rate (29%). Of those that were vitamin D insufficient (n = 235), most were Hispanic (51%), 36% White and 13% African American (see Table 1).

Vitamin D deficiency: HbA1c and BMI

There was no significant association between vitamin D deficiency and HbA1c levels (p = 0.108; see Table 1). However, there was a significant association between vitamin D deficiency and BMI (p = 0.035). When compared to obese children, non-obese children were less likely to be vitamin D deficient (71% vs 29%) (see Table 1).

Vitamin D deficiency: seasonal changes

Two hundred and eighty-six children (73%) had serum vitamin D levels collected in the summer months and 109 (25%) in the winter months. There was a significant and weak association between vitamin D status and season. In the summer, children were less likely to be vitamin D deficient (3% vs 6% in winter), less likely to be vitamin D insufficient (55% vs 71% in winter) and more likely to be vitamin D sufficient (55% vs 71% in winter), X2 (2) = 9.99, V = .16, p = 0.007.

Summer Months

For children that had vitamin D serum drawn in the summer, there were no significant associations of vitamin D deficiency based on sex. There was a significant association between vitamin D levels and age and race/ethnicity: Children in the deficient group were older (see Table 2).

**Table 2:** Participant Characteristics by Vitamin D Levels in Summer Months (March through October) N = 286; when percentages do not add to 100%, it is due to rounding. HbA1c: Hemoglobin A1c; BMI: Body mass index.

	Vitamin D deficient <15 ng/mL (n = 11)	Vitamin D insufficient 15-29 ng/mL (n = 158)	Vitamin D sufficient ≥30 ng/mL (n = 117)	P value
Sex				
Female	3 (27)	73 (46)	58 (49)	0.356
Male	8 (73)	85 (54)	59 (51)	
Age, years				
Mean (SD)	15.30 (1.83)	12.87 (3.20)	12.66 (3.32)	0.035
Median (Min-Max)	15.23 (12-18)	12.93 (3-18)	13.01 (3-18)	
Race				
African American	4 (36)	23 (15)	7 (6)	<0.001
Hispanic	2 (18)	79 (50)	37 (32)	
White	5 (46)	56 (35)	73 (62)	
HbA1C Status				
Adequate	5 (46)	76 (48)	74 (63)	0.038
Suboptimal	6 (54)	82 (52)	43 (37)	
BMI Status				
Obese	3 (27)	19 (12)	10 (9)	0.15
Non-Obese	8 (72)	139 (88)	107 (91)	

White children were more likely to be deficient than Hispanic children (46% vs 18%, respectively) and African American children (46% vs 36%). Moreover, African American children were more likely than Hispanic children to be vitamin D deficient (36% vs 18%, respectively). Of those that were vitamin D insufficient (n = 158), most were Hispanic (50%), 35% White and 15% African American (see Table 2).

In regards to the association between vitamin D deficiency and diabetes control, i.e. HbA1c levels, we found a significant association between vitamin D levels and HbA1c (p = 0.038). Additionally, a non-significant relationship between vitamin D levels and BMI was found (p = 0.038) (Table 2).

Winter Months

For children that had a serum vitamin D level drawn in the winter, there were no significant associations of vitamin D deficiency based sociodemographic factors. Additionally, no significant association was found between vitamin D deficiency and HbA1c levels (p = 0.081) or BMI (p = 0.080) in this subset (Table 3).

**Table 3:** Participant Characteristics by Vitamin D Levels in Winter Months (November through February) N = 109; when percentages do not add to 100%, it is due to rounding. HbA1c: Hemoglobin A1c; BMI: Body mass index.

	Vitamin D deficient <15 ng/mL (n = 6)	Vitamin D insufficient 15-29 ng/mL (n = 77)	Vitamin D sufficient ≥30 ng/mL (n = 26)	P value
Sex				
Female	5 (83)	44 (57)	10 (39)	0.086
Male	1 (17)	33 (43)	16 (61)	
Age, years				
Mean (SD)	13.32 (3.0)	13.13 (3.0)	12.53 (3.93)	0.691
Median (Min-Max)	14.29 (8-16)	13.33 (7-18)	13.84 (4-18)	
Race				
African American	1 (17)	7 (9)	3 (12)	0.54
Hispanic	3 (50)	41 (53)	9 (35)	
White	2 (33)	29 (38)	14 (54)	
HbA1C Status				
Adequate	1 (17)	41 (53)	9 (35)	0.081
Suboptimal	5 (83)	36 (47)	17 (65)	
BMI Status				
Obese	2 (33)	5 (7)	3 (12)	0.08
Non-Obese	4 (67)	72 (94)	23 (88)	

## Discussion

The data presented here shows that 64% of our pediatric patients with type 1 diabetes mellitus had a serum 25 (OH) vitamin D level below 30 ng/ml. These findings correlate with findings from other similar studies [14]. Moreover, the percentage of patients with type 1 diabetes mellitus in our study who had a serum 25 (OH) vitamin D level <30 ng/ml is comparable to rates reported in the NHANES Survey that evaluated the prevalence of vitamin D insufficiency in all US children (64% vs 70%) [13]. Our data suggests that vitamin D insufficiency and/or deficiency is significant but does not seem to be more prevalent in children with type 1 diabetes as compared to the general pediatric population.

Interestingly, the patients evaluated in our study reside in Central Florida, a region with high-intensity sunlight, high annual UVB exposure and year-round hot temperatures. The high incidence of vitamin D insufficiency documented in our subjects demonstrates that residing in areas with easy access to sunlight does not guarantee protection against the development of vitamin D deficiency or insufficiency.

Furthermore, among ethnic groups, 42% of our White children with type 1 diabetes were found to have a low 25 (OH) vitamin level. In contrast, only 29% of the African-Americans screened had a low 25 (OH) vitamin D level. Such differences are surprising since darker skin allows less UVB skin penetration resulting in decreased production of vitamin D. The prevalence of vitamin D insufficiency in Hispanic children was not considerably different from the African-American children with T1D (29%). Significant vitamin D deficiency was rare among all studied subjects and only 5% were found to have a vitamin D level lower than 15 ng/ml.

The importance of our findings is substantiated by the significant relationship between type 1 diabetes mellitus and vitamin D status. There has been extensive evidence in the role of vitamin D in many autoimmune conditions including T1D, rheumatoid arthritis, scleroderma, psoriasis and multiple sclerosis [15,16]. It has been demonstrated that vitamin D is important in the prevention of islet cell death and improvement in insulin production. Low vitamin D levels were shown to have a negative effect on beta-cell function [17]. Our overall study results revealed that poor glycemic control (evaluated by HbA1C) did not appear to be associated with the presence or severity of vitamin D deficiency. However, a closer look at the data by season revealed a significant association in the summer, but not in the winter. Although there is still intense debate about whether vitamin D supplementation in individuals with T1D that have vitamin D deficiency could lead to improvement in HbA1C levels, our data suggests that conflicting findings could be related to sample size and seasonal changes [18,19]. The recognition of significant prevalence of vitamin D insufficiency in this population also deserves careful consideration in terms of research linking low D levels with increased diabetes morbidity.

The need for a better understanding of the risk factors associated with development of comorbidities in patients with type 1 diabetes becomes clear when one analyzes its financial implications. Individuals with diagnosed diabetes mellitus, on average, have medical expenditures approximately two times higher than their peers without diabetes mellitus [20]. The annual cost of medical care for young people with diabetes is six times higher than medical care for children and teens without the disease, according to a U.S. Centers for Disease Control and Prevention study [21]. These numbers corroborate the need for implementing research that might further elucidate the mechanisms driving the rise in the incidence of pediatric T1D and what can be done to prevent long-term complications once the disease is established. Based on the escalating evidence of the role of vitamin D deficiency in the pathogenesis of diabetes and its complications, it becomes imperative to consider screening patients with T1D for vitamin D deficiency.

Currently, screening for the most frequently associated autoimmune conditions (i.e., thyroid dysfunction and celiac disease) in patients with T1D is a common practice and recommendation by the American Diabetes Association. Hypothyroidism due to autoimmune thyroiditis occurs in approximately 3-8% of children and adolescents with T1D [22]. Celiac disease occurs in 1-10% of these children and adolescents [23]. Among our patients with T1D, vitamin D insufficiency was significantly more common than the autoimmune conditions that are routinely screened for. Given the potential health implications and burden of untreated vitamin D deficiency in children with T1D and the ease of its treatment, we argue that pediatric endocrinologists should consider regularly screening their patients with T1D for vitamin D insufficiency/deficiency. Vitamin D supplementation is inexpensive and readily available and correction of vitamin D deficiency in individuals with T1D could potentially play a significant role in improving their health.

This is a retrospective study, thus it was not possible to match patients on important sociodemographic variables. Nonetheless, these data are a valuable contribution to the existing literature. Specifically, it is important to note that our sample is drawn from Central Florida. The Central Floridian population helps disregard the common belief that vitamin D deficiency/insufficiency is primarily due to patients residing in colder climates with low sunlight levels. Similar findings have been reported by other groups among patients living in a sun rich environment [24, 25]. Furthermore, the data values for vitamin D levels reported here are pre-supplementation, thereby eliminating the bias that supplementation could have on vitamin D status. Further research evaluating the role of vitamin D in T1D could bring benefits in treatment and potential prevention of type 1 diabetes mellitus in the pediatric population.

## Conclusions

The data from our study indicates that 64% of children with T1D in our practice had low vitamin D levels, similar to the general US population. Interestingly, vitamin D deficiency is still quite prevalent in the sun rich environment of central Florida. We noted significant differences based on ethnicity, BMI and seasonal variation. Vitamin D deficiency was more common among White children as compared to Hispanic children (42% vs 29%, respectively) and African American children. When compared to obese children, non-obese children were less likely to be vitamin D deficient (71% vs 29%). In the summer, children were less likely to be vitamin D deficient or vitamin D insufficient.

A link between autoimmune conditions and vitamin D deficiency/insufficiency has been implicated. Considering the high incidence of vitamin D deficiency among children with T1D, its potential health implications and its ease of treatment, we argue that pediatric endocrinologists should consider regularly screening their patients with T1D for vitamin D insufficiency/deficiency.
